# Viral Paratransgenesis in the Malaria Vector *Anopheles gambiae*

**DOI:** 10.1371/journal.ppat.1000135

**Published:** 2008-08-22

**Authors:** Xiaoxia Ren, Egbert Hoiczyk, Jason L. Rasgon

**Affiliations:** 1 The Johns Hopkins Malaria Research Institute, Baltimore, Maryland, United States of America; 2 The W. Harry Feinstone Department of Molecular Microbiology and Immunology, Bloomberg School of Public Health, Johns Hopkins University, Baltimore, Maryland, United States of America; Stanford University, United States of America

## Abstract

Paratransgenesis, the genetic manipulation of insect symbiotic microorganisms, is being considered as a potential method to control vector-borne diseases such as malaria. The feasibility of paratransgenic malaria control has been hampered by the lack of candidate symbiotic microorganisms for the major vector *Anopheles gambiae*. In other systems, densonucleosis viruses (DNVs) are attractive agents for viral paratransgenesis because they infect important vector insects, can be genetically manipulated and are transmitted to subsequent generations. However, *An. gambiae* has been shown to be refractory to DNV dissemination. We discovered, cloned and characterized the first known DNV (AgDNV) capable of infection and dissemination in *An. gambiae*. We developed a flexible AgDNV-based expression vector to express any gene of interest in *An. gambiae* using a two-plasmid helper-transducer system. To demonstrate proof-of-concept of the viral paratransgenesis strategy, we used this system to transduce expression of an exogenous gene (enhanced green fluorescent protein; EGFP) in *An. gambiae* mosquitoes. Wild-type and EGFP-transducing AgDNV virions were highly infectious to *An. gambiae* larvae, disseminated to and expressed EGFP in epidemiologically relevant adult tissues such as midgut, fat body and ovaries and were transmitted to subsequent mosquito generations. These proof-of-principle data suggest that AgDNV could be used as part of a paratransgenic malaria control strategy by transduction of anti-*Plasmodium* peptides or insect-specific toxins in *Anopheles* mosquitoes. AgDNV will also be extremely valuable as an effective and easy-to-use laboratory tool for transient gene expression or RNAi in *An. gambiae*.

## Author Summary

Paratransgenesis, the genetic manipulation of mosquito symbiotic microorganisms, is being considered as a potential strategy to control malaria. Microorganisms associated with *Anopheles* mosquitoes could be manipulated to alter the mosquito's ability to become infected with and transmit the malaria parasites, or reduce mosquito fecundity or lifespan. We identified the first potential microorganism (*An. gambiae* densovirus; AgDNV) for paratransgenesis of the major malaria vector *Anopheles gambiae*. AgDNV is highly infectious to *An. gambiae* larvae, disseminates to adult tissues and is transmitted vertically to subsequent generations. Recombinant AgDNV was able to transduce expression of an exogenous gene (EGFP) in *An. gambiae* cells and mosquitoes. EGFP-transducing virions infected mosquitoes, expressed EGFP in epidemiologically relevant tissues and were transmitted to offspring in a similar manner to wild-type virus. AgDNV could be used as part of a paratransgenic malaria control strategy by transduction of anti-*Plasmodium* genes or insect-specific toxins in *Anopheles* mosquitoes, as well as an easy-to-use system for transient gene expression and RNAi for basic laboratory research.

## Introduction

Transmitted by *Anopheles* mosquitoes, malaria is a disease responsible for inordinate mortality, morbidity and economic loss worldwide [1]. Failure of traditional control methodologies has stimulated efforts to develop novel genetic strategies to control the mosquito vectors, particularly *An. gambiae*. Transgenic manipulation of *An. gambiae* has proven to be especially challenging, with few published successes [2]–[3]. Paratransgenesis, the genetic manipulation of insect symbiotic microorganisms, is being considered as an alternative to traditional transgenic strategies [4]–[5]. Microorganisms associated with *Anopheles* could be manipulated to alter the mosquito's ability to become infected with and transmit the malaria parasites, or reduce mosquito fecundity or lifespan. A suitable microbial candidate for paratransgenic malaria control would have a symbiotic (mutualistic, commensal or parasitic) relationship with the vector, be readily propagated and stably engineered to express the gene(s) of interest without compromising microorganism fitness, and be easily delivered to wild mosquito populations [4]. Ideally, the engineered microbe would also be maintained in the environment, be passed to subsequent mosquito generations and have limited effects on non-target species.

Densonucleosis viruses, or “densoviruses” (DNVs), are non-enveloped single-stranded DNA icosahedral viruses in the family Parvoviridae (subfamily Densovirinae) that infect arthropods such as mosquitoes. Mosquito DNVs have narrow host ranges and are maintained in natural populations by a cycle that includes both horizontal and vertical transmission from infected adults to larvae. DNVs possess some of the smallest known viral genomes (4–6 kb), a trait that makes them highly amenable as molecular tools because the entire genome can be placed into an infectious plasmid, manipulated by standard cloning techniques, and used to express foreign genes (i.e. anti-parasite or toxin) upon infection in cell cultures or live mosquitoes [6]. DNV infectious clones, expression systems, and lethal biocontrol agents (based on the *Aedes aegypti* densovirus; AeDNV) have been developed and show promise for *Aedes* mosquitoes [6]–[8]. When injected into larvae, AeDNV virions can infect *An. gambiae* [9], but when infection by larval exposure to virions is attempted, AeDNV does not disseminate in *An. gambiae* [8]. Similar results were observed when researchers could only infect *An. gambiae* with TaDNV (isolated from a *Toxorhynchites amboinensis* cell line) by adult injection but not larval exposure [10]. Thus, DNVs have previously not been considered useful for paratransgenic manipulation or control of *An. gambiae*.

We serendipitously discovered a novel DNV capable of infection and dissemination in *An. gambiae* larvae (AgDNV) while investigating a PCR artifact in an unrelated experiment. AgDNV is highly infectious to *An. gambiae* larvae, disseminates to adult tissues, and is passed on to subsequent generations. Recombinant AgDNV genomes were able to transduce expression of an exogenous transgene (enhanced green fluorescent protein; EGFP) in cultured *An. gambiae* cells and mosquitoes and were transmitted to subsequent mosquito generations. AgDNV will form the foundation for the development of much-needed tools for routine manipulation of *An. gambiae* and paratransgenic malaria control.

## Results/Discussion

**Figure 1:**
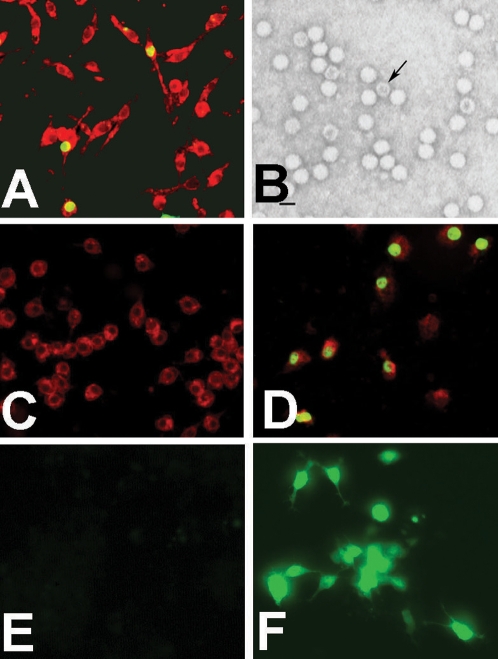
Visualization of densovirus infections in cultured *An. gambiae* cells. In IFA's, cells were counterstained with Evan's Blue (red fluorescence) to visualize cell morphology. A: wild-type AgDNV visualized in Sua5B cells by IFA (IFA positive control); B: Negative-stain TEM of AgDNV particles purified from Sua5B cell. Arrow denotes unpackaged virion. Bar = 20 µm. C: Lack of natural densovirus infection in Moss55 cells (IFA negative control); D: densovirus visualized in Moss55 cells by IFA after transfection with pBAg; E: live-cell epifluorescence showing no fluorescence in untransfected Moss55 cells (epifluorescence negative control); F: live-cell epifluorescence showing cytoplasmic EGFP expression in Moss55 cells transfected with pBAg and pAgActinGFP.

In the course of verifying *Wolbachia* infection of *An. gambiae* cell line Sua5B [11], we observed a weak band at approximately 400 bp instead of the expected ∼600 bp fragment using the putatively *Wolbachia*-specific primers 81F and 691R [12]. We isolated the band from the gel for cloning and sequencing. We compared the 358 bp sequence to the BLAST database where it hit with high homology (87%) to a portion of the NS1 gene of the *Aedes aegypti* densovirus (AeDNV) (GenBank #M37899) [13], indicating that there was a DNV present in our *Anopheles* cell culture which we termed AgDNV. We used a densovirus-specific immunofluorescence assay (IFA) to visualize AgDNV infection in Sua5B cells, which confirmed localized AgDNV infection in cell nuclei [6] (Figure 1A). We then determined that AgDNV virions isolated from Sua5B cells were highly infectious to *An. gambiae* larvae *in vivo*. In order to evaluate both viral infection efficiency and lethality, we infected naïve first instar larvae (Keele strain) by either allowing larvae to feed on infected Sua5B cell cultures or by adding filtered infected Sua5B cell lysate to the larval rearing water. Both methods resulted in similarly high infection levels in emerging adults as determined by PCR (whole cells: 62%, N = 39; lysate: 57%, N = 53; Fishers Exact *P* = 0.67). Quantitative PCR indicated that larvae were exposed to approximately 2.1×10^11^±0.97×10^11^ viral genome equivalents per ml, which is well within the range that causes significant mortality for other DNV isolates [14]. However, we observed no difference in survival to adulthood between the controls and either infection treatment (control: 34%, N = 50; whole cells: 26%, N = 150, lysate: 35%, N = 150, chi-square = 3.27, d.f. = 2, *P* = 0.195), possibly due to adaptation of the virus to cell culture conditions.

**Figure 2:**
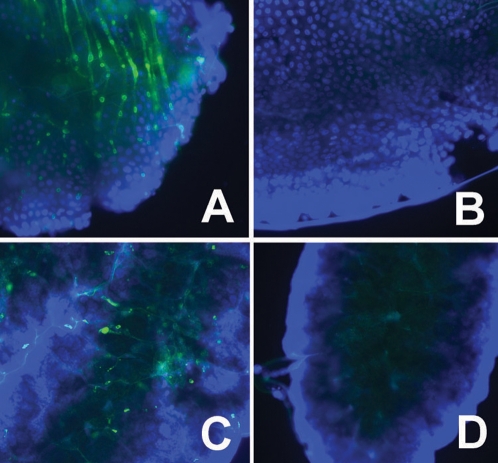
IFA detection of AgDNV infection of dissected adult tissues. Mosquitoes were infected as first-instar larvae with AgDNV purified from Sua5B cells. A, B: midgut; C, D: ovary. A, C: infected with AgDNV; B, D: control. Blue: cell nuclei stained with DAPI, green: densovirus.

To test for transtadial transmission and dissemination of AgDNV in adult mosquitoes, we infected first-instar *An. gambiae* larvae, transferring them to clean virus-free water after 2 days. Uninfected control larvae were exposed to culture media. After adult emergence, we dissected adult tissues and performed densovirus-specific immunofluorescence microscopy. AgDNV clearly disseminates and infects adult midgut and ovary (Figure 2). We then assessed whether AgDNV could be transmitted to subsequent generations. We treated mosquitoes for 24 hours as larvae with AgDNV, which were reared to adulthood, bloodfed, allowed to oviposit, and their offspring reared to adulthood and assayed for AgDNV by PCR. Fifty percent of treated mosquitoes were positive for virus by PCR (N = 42). Twenty-eight percent (N = 71) of their offspring were positive for infection, indicating that AgDNV was transmitted between generations, either by vertical transmission or by horizontal transmission from adults to larvae.

**Figure 3:**
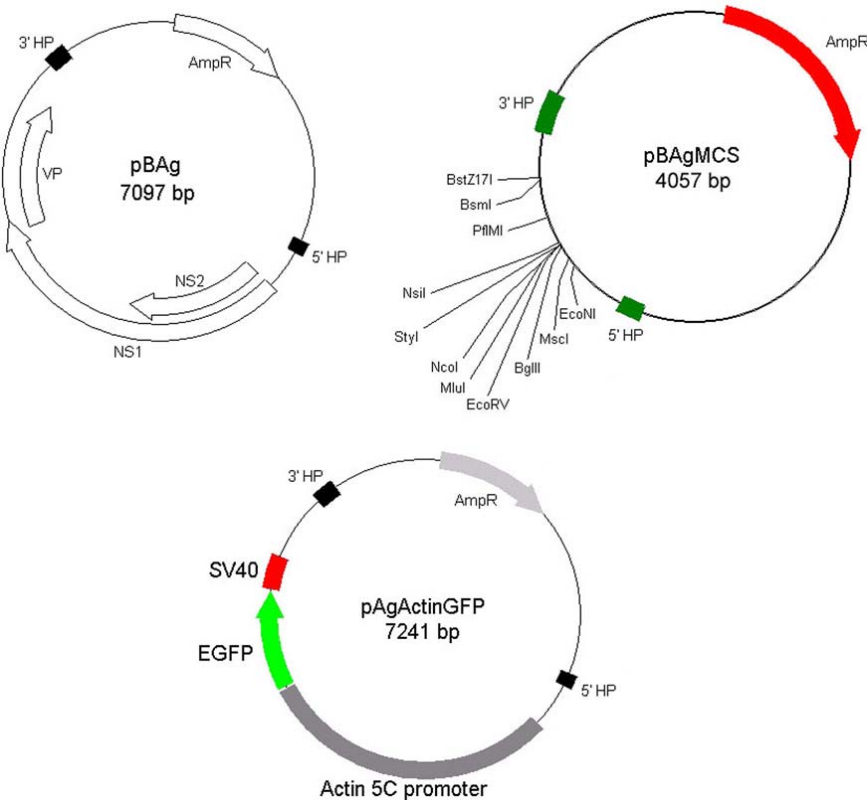
Simplified plasmid schematics for cloned AgDNV genome (pBAg), expression vector (pBAgMCS) and reporter (pAgActinGFP). HP: hairpin; VP: viral capsid protein; NS1, NS2: non-structural proteins 1 and 2; AmpR: ampicillin resistance. Full plasmid sequences are available as supplementary material (Text S1).

**Figure 4:**
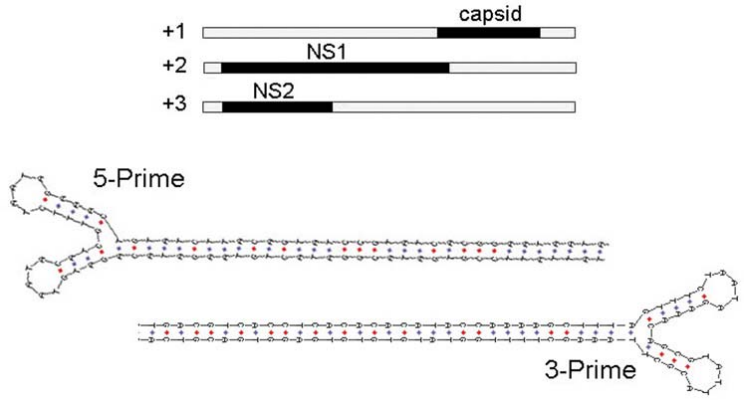
AgDNV genome organization. Top: AgDNV predicted open reading frames. ORF's overlap one another and each is in a different reading frame. Bottom: predicted 5-prime and 3-prime AgDNV terminal hairpin structures.

**Figure 5:**
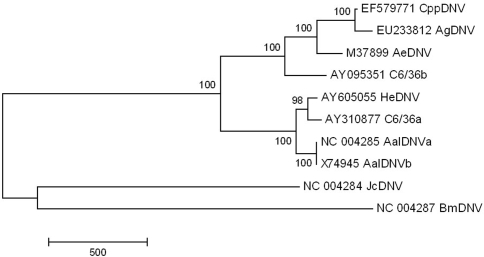
Maximum parsimony phylogenetic tree of DNVs based on complete viral genome sequences (CI = 0.92, RI = 0.84). Tree was generated using MEGA v.4. Taxon codes represent GenBank accession numbers. Numbers at nodes represent bootstrap support values (500 replicates). Scale bar represents number of nucleotide differences.

To purify AgDNV particles for microscopy and isolation of the viral genome, we fractionated crude Sua5B cell lysates in a cesium chloride gradient and examined fractions for viral particles by negative-stain transmission electron microscopy. We isolated numerous icosahedral, non-enveloped particles of the expected size (20 nm) (Figure 1B). We extracted the viral DNA from this gradient fraction and cloned the entire viral genome into the pBluescript S/K(-) cloning vector (denoted pBAg; Figure 3, Text S1). The cloned AgDNV genome is typical of mosquito DNVs. It is 4139 nt (GenBank #EU233812) in length and has 3 overlapping reading frames: the viral capsid and 2 non-structural (NS) proteins. The 5-prime and 3-prime ends of the genome consist of inverted hairpin repeats and are predicted to fold into perfect Y-shaped hairpin structures (Figure 4). Phylogenetic analysis of the entire AgDNV genome indicated that AgDNV falls within the “Asian” clade of known mosquito densoviruses [6]. Within the coding region, it is most closely related to a recently-described cluster of DNVs isolated from *Culex pipiens pallens* (CppDNV) in China [15] (Figure 5).

To confirm infectiousness of pBAg to *An. gambiae* cells, we transfected it into the *An. gambiae* cell line Moss55 (which lacks endogenous densovirus infection; Figure 1C) and observed DNV-specific signal in transfected cell nuclei by IFA (Figure 1D). However, when purified from the cell culture, virions produced from pBAg were unable to infect *An. gambiae* larvae *in vivo*. By sequencing fragments of directly-cloned viral DNA isolated from Sua5B cells, we identified multiple clones with point mutations in the 5-prime UTR and non-synonymous point mutations in the NS1 and NS2 genes (Table 1), suggesting that AgDNV was not homogeneous within Sua5B cells, but rather exists as a heterogeneous population of viral genomes that may differ in their ability to infect *Anopheles* larvae. To select for the viral genotype(s) that were infectious to *An. gambiae* larvae, we infected larvae as first-instars with virus isolated from Sua5B cells, reared them to adulthood and sequenced most of the coding portion of the AgDNV genome (nucleotides 403–3709) from 5 infected females. All 5 sequences were identical, indicating that within the viral population in Sua5B cells only one genotype was infectious to larvae. This genotype differed from pBAg at 3 sites: A636G (Lys to Glu in NS1), A1174C (Asp to Ala in NS1 and Ile to Leu in NS2) and A3399T, (Asn to Ile in capsid) (no synonymous mutations were detected). We used site-directed mutagenesis to reproduce these three mutations in pBAg (denoted pBAgα). Virions produced from pBAgα in Moss55 cells had similar infectivity to *An. gambiae* larvae as wild-type AgDNV from Sua5B cells as determined by both PCR and IFA.

**Table 1:** Additional AgDNV SNPs identified from directly-cloned and sequenced fragments of AgDNV DNA isolated from Sua5B cells.

	5′ UTR SNPs			Non-synonymous SNPs			
Site	201	269	283	458	1045	1094	1395
Clone	C	A	G	A	G	C	A
55	T	G					
57	T	G					
58						T^2^	
104				G^1^			
105							T^4^
106					A^3^		
119		G	A				
120		G	A				
125	T	G					

12 additional clones were identified with the same sequence as pBAg.

1 N to S in NS2.

2 A to V in NS2.

3 S to N in NS1; A to T in NS2.

4 Q to L in NS1.

We used pBAg to create a flexible gene transduction construct by deleting most of the viral genome between the hairpin sequences and inserting a multiple cloning site (pBAgMCS; Figure 3, Text S1). Using pBAgMCS, we can easily construct viral transducing genomes carrying any gene-promoter combination of interest, and by supplying the missing viral proteins *in trans* with pBAgα or wild-type virus, we can express the gene in *An. gambiae* mosquitoes simply by adding the virions to the larval rearing water. As proof-of-concept, we inserted the enhanced green fluorescent protein (EGFP) under control of the constitutive *Drosophila* actin5C promoter into the multiple cloning site of pBAgMCS (pAgActinGFP; Figure 3, Text S1). When pBAgα and pAgActinGFP were simultaneously transfected into Moss55 cells, we observed cytoplasmic EGFP expression 24–48 hours post-infection (Figure 1E, F). We observed fluorescent cells in the culture even after 10 passages (approximately 2 months), indicating that the helper and transducing virions were replicating in the cells. We do not believe that these results are due to integration of the viral genome into the host genomic DNA, as integration is not known to occur for DNVs in the genus Brevidensovirus (the genus AgDNV belongs to), although integration does occur for other DNV genera [6].

**Figure 6:**
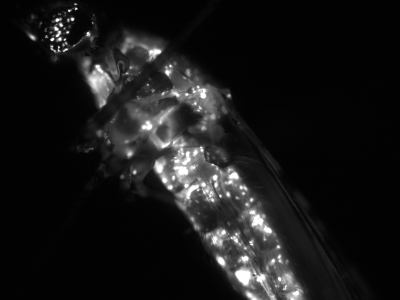
Fully-disseminated EGFP expression in *An. gambiae* adult infected as larvae with a mix of wild-type (pBAgα) and EGFP-transducing (pAgActinGFP) virions.

**Figure 7:**
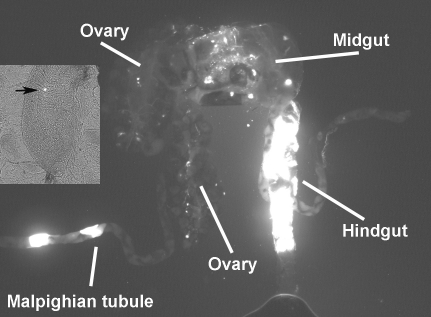
EGFP expression in dissected tissues of adult *An. gambiae* female. Fluorescence is observable in the midgut, hindgut, ovaries and malpighian tubules. Inset: EGFP-positive inclusion in mature oocyte (arrow).

We purified helper and EGFP-transducer virions from transfected Moss55 cells, exposed first-instar *An. gambiae* larvae to them and assayed emerged adults for EGFP expression by fluorescence microscopy. EGFP expression was observed in approximately 50% of adults (N>100). We observed similar results when virus from Sua5B cells rather than pBAgα was used as helper. EGFP expression was first observed in the fat body, later disseminating to other tissues such as the eye, midgut, hindgut, malpighian tubules and ovaries (Figures 6 and 7).

**Figure 8:**
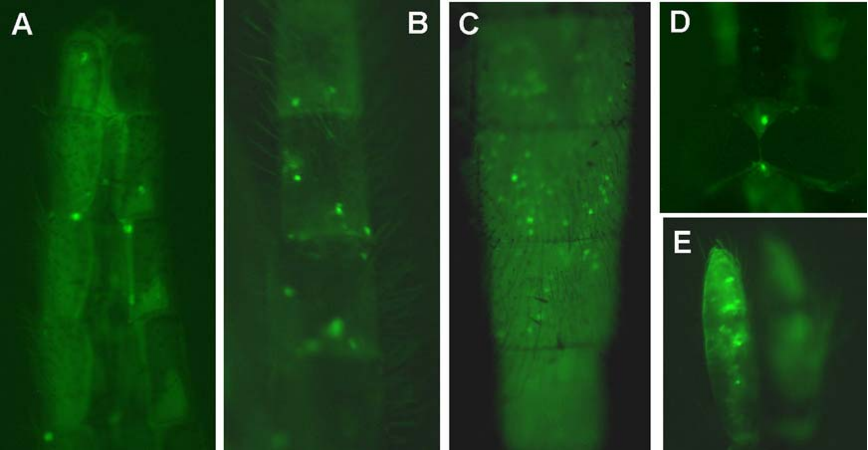
EGFP expression in *An. gambiae* F1 adults infected with wild-type AgDNV (from pBAgα) and EGFP-transducing virions (from pAgActinGFP), which demonstrates transmission of transducing virions between mosquito generations. A–C: EGFP expression in abdomen, D: EGFP expression in head, E: EGFP expression in maxillary palp.

EGFP-positive mosquitoes were allowed to reproduce. We observed EGFP expression in approximately 20% of F1 offspring (N>50, Figure 8) and detected EGFP DNA by PCR and sequencing from EGFP-expressing F1 mosquitoes (N = 8). We continued to breed the offspring and again assessed EGFP expression in the F3 generation, where 20% of the mosquitoes had observable EGFP fluorescence (N = 20). These data indicate that AgDNV can be used to drive expression of exogenous transgenes in *An. gambiae* and that transducing virions are transmitted to subsequent generations, similar to wild-type virus. While it is not clear at this point whether offspring are infected by transovarial transmission or horizontal transmission from adults to larvae, we detected EGFP in both developing ovarioles and in mature oocytes (Figure 7) suggesting that transovarial transmission may be involved.

The development of novel, efficacious malaria control methods is critical to reduce the enormous public health and economic burdens experienced in affected areas. Densovirus-based tools for control of *Anopheles* mosquitoes are very attractive for this purpose due to their specificity, stability, ease in engineering, ability to spread horizontally and vertically and accumulate in natural environments, and recent advances in large-scale production and purification methods [16]–[17]. Recombinant AgDNV could potentially be used to control malaria by transduction in *An. gambiae* of anti-*Plasmodium* peptides to block parasite transmission or insect-specific toxins to reduce mosquito population density or mosquito lifespan. AgDNV will also be extremely valuable as an effective and easy to use laboratory tool for transient gene expression or RNAi [6] in *An. gambiae*.

## Materials and Methods

### Mosquito rearing

The *Anopheles gambiae* Keele strain was used for experiments in 30 cm cube cages kept in a walk-in insectary at 28°C and 80% relative humidity. Mosquitoes were allowed access to a cotton wick soaked in 20% sucrose as a carbohydrate source. Adults were allowed to bloodfeed on an anesthetized mouse 5 days post-emergence. Two days after bloodfeeding, an oviposition substrate (consisting of a filter paper cone inside a 50 ml beaker half-filled with water) was introduced into cages and filter papers containing eggs removed the next day, placed into a 41×34×6 cm rearing tray half-filled with distilled water and one pellet dry cat food, with one additional food pellet added daily after day 3. Pupae were picked with an eye-dropper, placed in a cup and introduced into cages (∼200 pupae/cage) to begin the next generation.

### Mosquito cell maintenance and transfection

The *Anopheles gambiae* cell lines Sua5B and Moss55 were grown at room temperature in Schneider's medium (Sigma) supplemented with 10% fetal bovine serum. DNAs used for transfection were prepared using a QIAGEN Plasmid Purification Kit (Qiagen, Valencia, CA) according to the manufacturer's protocol. For the transfection of cells with different plasmids, one µg of total plasmid DNA (0.5 µg vector and 0.5 µg helper) was used with Effectene® Transfection Reagent (QIAGEN) according to the manufacturers suggested protocol.

### PCR amplification of AgDNV DNA

Genomic DNA was extracted from Sua5B cells using DNEasy kits (QIAGEN, Valencia, CA) according to the manufacturer's suggested protocol. Unexpected PCR amplification of an approximately 400-bp fragment of AgDNV was amplified using *Wolbachia* primers wsp81F (5′-TGG-TCC-AAT-AAG-TGA-TGA-AGA-AAC-3′) and wsp691R (5′-AAA-AAT-TAA-ACG-CTA-CTC-CA-3′) [12]. PCR amplicons were separated by 1% agarose gel electrophoresis, stained with ethidium bromide, and visualized with UV light. PCR fragments were cloned into the pCR4-TOPO vector and sequenced. We detected AgDNV infection in infected mosquitoes using primers DensoVF (5′-CAG-AAG-GAT-CAG-GTG-CAG-3′) and DensoVR (5′-GCT-ACT-CCA-AGA-GCT-ACT-C-3′) using Sua5B as a positive control and water as a negative control.

### Immunofluorescence assay

Cells were grown overnight in 8-well chamber slides, then fixed with 4% paraformaldehyde. Fixed cells were washed 3 times with PBS, permeabilized with 0.01% Triton X-100 in PBS, and washed 3 times in PBS. Cells were incubated in 1% BSA, PBS pH 7.4 for 30 min to block non-specific antibody binding. Cells were incubated with primary antibody (1∶1000) in 1% BSA, PBS pH 7.4 for 60 min and washed for 10 minutes three times with PBS pH 7.4. Cells were incubated with goat anti-rabbit IgG FITC conjugate (Sigma) (1∶500), Evans Blue (1∶1000), in 1% BSA, PBS pH 7.4 for 60 min at RT, then washed for 10 minutes three times with PBS pH 7.4. Cells were stained with DAPI, mounted and visualized by epifluorescent microscopy.

### Infection of mosquito larvae with AgDNV

First-instar larvae were either introduced directly into culture flasks containing Sua5B cells or were infected by adding Sua5B cell lysate to the rearing water. In this case, Sua5B cells were pelleted in a 50 ml conical tube by centrifuging for 10 minutes at 2,500 G, 4°C. The pellet was resuspended in 20 ml PBS. Cells were lysed by vortexing with sterile 3 mm borosilicate glass beads for 5 minutes. Approximately 20 ml cell lysate was added to 20 ml ddH_2_0 with approximately 50 first-instar *An. gambiae* larvae Keele strain (4 replicates). Larvae were exposed to virus for 24 hours, then were transferred to clean water with larval food.

### Intergenerational transfer of AgDNV

First-instar larvae were infected with Sua5B lysate, reared to adulthood, allowed to bloodfed on an anesthetized mouse approximately one week post-emergence, and offspring produced as described above. Adults and offspring were tested for AgDNV by PCR using primers DensoVF and DensoVR, using Sua5B as a positive control and water as a negative control.

### Purification of AgDNV particles

Sua5B cells were pelleted and lysed as described above. The supernatant was removed to a new tube and cellular debris pelleted by centrifuging for 20 minutes at 10,000 G, 4°C. The supernatant was centrifuged at 35,000 rpm for 75 minutes, 4°C to pellet virion particles. The virion pellet was removed and further purified by 1 M sucrose cushion centrifugation for 120 minutes at 39,000 rpm, 4°C. The final pellet was fractionated in a CsCl (0.3 g/ml) gradient at 60,000 rpm overnight at 8°C. The virion band was removed from the gradient for DNA extraction and TEM.

### TEM

Purified virus particles were applied to glow-discharged carbon-coated grids and negatively stained with 2% (w/v) uranyl acetate. Electron micrographs were recorded on Kodak SO-163 film using a Philips CM12 electron microscope at nominal magnifications of 37,000× to 52,000×.

### Isolation of AgDNV DNA

Pure virion particles isolated from the gradient were incubated in 300 µl buffer (100 mM EDTA, 10 mM Tris-HCl, 0.1% SDS, 100 µg/ml proteinase K, pH 8.0) overnight at 55°C. The next day, the mixture was centrifuged at 14,000 rpm for 2 minutes to pellet debris. DNA was extracted from the supernatant twice using 1 volume of phenol∶chloroform (1∶1). One tenth volume of 3 M sodium acetate and 2.5 volumes of cold ethanol were added to precipitate viral DNA. DNA was pelleted by centrifugation at 14,000 G for 20 minutes, washed with 70% cold ethanol, air dried and resuspended in 5 µl 10 mM Tris-HCl (pH 8.5).

### Cloning the AgDNV genome (pBAg)

600 ng AgDNV genomic DNA was blunt-ended by incubating for 15 minutes at room temperature with 10 units Klenow fragment. Viral DNA was ethanol precipitated, cloned into the EcoRV site of plasmid pBluescript S/K(-) and transformed into SURE® competent cells (Stratagene). 20 clones were selected and sequenced to confirm viral inserts. We were unable to clone the entire AgDNV genome in one step, and thus assembled the genome from two clones that, together, contained the entire AgDNV genome. These clones were digested with NcoI and XbaI and ligated together to build a full-length infectious clone (pBAg). pBAg infectivity in Moss55 cells was confirmed by transfection and IFA as described.

### qPCR for virus quantification

pBAg plasmid was used as a copy-number standard for viral genome quantification as previously described [14]. The plasmid has an estimated mass of 7.78×10^−18^ g/copy. Plasmid concentrations were determined using an ND-1000 NanoDrop spectrophotometer (Thermo Fisher Scientific), and serial dilutions were made from 50 µM to 5×10^−8^ µM to generate a standard curve that ranged from 6.4×10^10^ viral genome equivalents/µL (geq/µL) to 6.4×10^0^ geq/µL in ten-fold increments. Primers were designed based on regions within the overlapping NS1 and NS2 genes that were highly conserved amongst all known mosquito densovirus isolates, as previously described [14]. The forward primer (5′-CAT-ACT-ACA-CAT-TCG-TCC-TCC-ACA-A-3′) and reverse primer (5′-CTT-GGT-GAT-TCT-GGT-TCT-GAC-TCT-3′) produce an 183 bp amplicon. The Quantitect SYBR Green Kit (Qiagen) was used in a 25 µL reaction containing 0.3 µM of each primer, and 5 µL of a 1/100 dilution of the Sua5B viral infection prep. Real-time PCR was performed on an ABI Prism model 7300 using 96-well reaction-plates (ABI) and MicroAmp Optical Adhesive Film (ABI) with a program of: (1) 50°C for 2 min, (2) 95°C for 15 min, (3) 45 cycles of i) 94°C for 15 sec, ii) 55°C for 30 sec , iii) 72°C for 30 sec. Data was collected each cycle at step 3iii, and the 45^th^ cycle was followed by a dissociation program to verify specific amplification.

### Constructing an infectious cloned AgDNV genome (pBAgα)

Virions produced by pBAg were not infectious to *An. gambiae* larvae. We infected larvae with virus isolated from Sua5B cells, reared larvae to adulthood and screened for infected mosquitoes by PCR as described. We selected 5 individual infected mosquitoes, sequenced the coding region of the virus that infected them and identified 3 mutations that all had in common as described in the text. We reproduced these mutations in pBAg by site-directed mutagenesis using the QuikChange Multi-Site Directed Mutagenesis Kit (Stratagene) with the manufacturer's protocol.

### AgDNV-based expression vector (pBAgMCS)

pBAgMCS carries a multiple cloning site (MCS) flanked by the 5-prime and 3-prime AgDNV hairpin sequences. The MCS possesses 5 common unique cloning sites: NsiI, NcoI, MluI, EcoRV and BglII (and several other less common cut sites, Figure 5). NsiI, NcoI, MluI, and BglII produce sticky ends for directional subcloning, while EcoRV produces blunt ends for blunt-end ligation procedures. We used pBAg as template for PCR using primers MCSF3 (5′- CCC-AAA-CCT-ATA-TAA-GGC-AAC-TGG-AAT-CGA-AGG-A -3′) and MCSR2 (5′- CCA-ATG-CAT-CCA-TGG-ACG-CGT-GAT-ATC-AGA-TCT-TGT-ATT-GTC-TCG-GTG-CA-3′) to amplify part of the 3-prime UTR, adding the MCS to the amplicon as part of the primer. The resultant product and pBAg were double-digested with NsiI and EcoNI. The digested pBAg was CIP-treated to prevent autoligation, and the 2 products ligated together with T4 ligase. The construct was transformed into SURE Competent cells (Stratagene), clones screened and proper vector construction confirmed by sequencing.

### EGFP transducing vector (pAgActinGFP)

The actin5C-EGFP-SV40 cassette was PCR-amplified from pHermes[act5C:EGFP] using primers Actin5CegfpF (5′-CCC-AAA-GAT-ATC-CGA-TCG-CTC-CAT-TCT-TG-3′) and Actin5CegfpR (5′-CCC-AAA-GAT-ATC-CGC-TTA-CAA-TTT-ACG-CC-3′) using *pfuUltra* II Fusion HS DNA Polymerase (Stratagene) with the manufacturers suggested protocol. The PCR product was digested with EcoRV. pBAgMCS was digested with EcoRV, and CIP-treated to prevent autoligation. The 2 products were ligated together with T4 ligase and the construct transformed into SURE competent cells. Clones were screened and proper insert confirmed by sequencing.

### EGFP transduction in *Anopheles gambiae* cells and mosquitoes

A combination of pBAgα and pAgActinGFP were transfected into Moss55 cells (or pAgActinGFP into Sua5B cells) as described. EGFP expression was monitored by fluorescence microscopy daily beginning 24 hours post-transfection. For mosquito infections, virion particles were purified from cells 1–2 weeks post-transfection by glass bead lysis/filtration and first-instar larvae infected directly as described above. EGFP expression in cells, dissected tissues and mosquitoes was monitored using an Olympus BX41 epifluorescent compound microscope. Images were captured using a Macrofire monochrome digital camera (Optronics).

### Intergenerational transfer of EGFP-transducing AgDNV

Mosquitoes which had observable EGFP expression were allowed to oviposit, offspring reared and EGFP expression in offspring assessed as described above. DNA was extracted from positive offspring and EGFP DNA detected using primers egfpF2 (5′-TGA-AGT-TCA-TCT-GCA-CCA -3′) and egfpR2 (5′-CAG-CAG-GAC-CAT-GTG-ATC-3′). PCR was conduced using pAgActinGFP as a positive control and water as negative control. Amplicons were gel purified and directly sequenced.

## Supporting Information

Text S1 Full sequences for constructs outlined in manuscript
(0.04 MB DOC)
