# Supplementary material for: Viral Paratransgenesis in the Malaria Vector Anopheles gambiae
Source: PLoS Pathog. 2008 Aug 22;4(8):e1000135. doi: 10.1371/journal.ppat.1000135 (PMC2500179; doi:10.1371/journal.ppat.1000135)
Supplement: Text S1 — Full sequences for constructs outlined in manuscript (0.04 MB DOC) [file ppat.1000135.s001.doc]

Ren et al.

Construct sequences:

Full sequence of pBAg (7097bp) (yellow denotes virus insert, red denotes changed nucleotides in pBAgα):

GCACTTTTCGGGGAAATGTGCGCGGAACCCCTATTTGTTTATTTTTCTAAATACATTCAAATATGTATCCGCTCATGAGACAATAACCCTGATAAATGCTTCAATAATATTGAAAAAGGAAGAGTATGAGTATTCAACATTTCCGTGTCGCCCTTATTCCCTTTTTTGCGGCATTTTGCCTTCCTGTTTTTGCTCACCCAGAAACGCTGGTGAAAGTAAAAGATGCTGAAGATCAGTTGGGTGCACGAGTGGGTTACATCGAACTGGATCTCAACAGCGGTAAGATCCTTGAGAGTTTTCGCCCCGAAGAACGTTTTCCAATGATGAGCACTTTTAAAGTTCTGCTATGTGGCGCGGTATTATCCCGTATTGACGCCGGGCAAGAGCAACTCGGTCGCCGCATACACTATTCTCAGAATGACTTGGTTGAGTACTCACCAGTCACAGAAAAGCATCTTACGGATGGCATGACAGTAAGAGAATTATGCAGTGCTGCCATAACCATGAGTGATAACACTGCGGCCAACTTACTTCTGACAACGATCGGAGGACCGAAGGAGCTAACCGCTTTTTTGCACAACATGGGGGATCATGTAACTCGCCTTGATCGTTGGGAACCGGAGCTGAATGAAGCCATACCAAACGACGAGCGTGACACCACGATGCCTGTAGCAATGGCAACAACGTTGCGCAAACTATTAACTGGCGAACTACTTACTCTAGCTTCCCGGCAACAATTAATAGACTGGATGGAGGCGGATAAAGTTGCAGGACCACTTCTGCGCTCGGCCCTTCCGGCTGGCTGGTTTATTGCTGATAAATCTGGAGCCGGTGAGCGTGGGTCTCGCGGTATCATTGCAGCACTGGGGCCAGATGGTAAGCCCTCCCGTATCGTAGTTATCTACACGACGGGGAGTCAGGCAACTATGGATGAACGAAATAGACAGATCGCTGAGATAGGTGCCTCACTGATTAAGCATTGGTAACTGTCAGACCAAGTTTACTCATATATACTTTAGATTGATTTAAAACTTCATTTTTAATTTAAAAGGATCTAGGTGAAGATCCTTTTTGATAATCTCATGACCAAAATCCCTTAACGTGAGTTTTCGTTCCACTGAGCGTCAGACCCCGTAGAAAAGATCAAAGGATCTTCTTGAGATCCTTTTTTTCTGCGCGTAATCTGCTGCTTGCAAACAAAAAAACCACCGCTACCAGCGGTGGTTTGTTTGCCGGATCAAGAGCTACCAACTCTTTTTCCGAAGGTAACTGGCTTCAGCAGAGCGCAGATACCAAATACTGTCCTTCTAGTGTAGCCGTAGTTAGGCCACCACTTCAAGAACTCTGTAGCACCGCCTACATACCTCGCTCTGCTAATCCTGTTACCAGTGGCTGCTGCCAGTGGCGATAAGTCGTGTCTTACCGGGTTGGACTCAAGACGATAGTTACCGGATAAGGCGCAGCGGTCGGGCTGAACGGGGGGTTCGTGCACACAGCCCAGCTTGGAGCGAACGACCTACACCGAACTGAGATACCTACAGCGTGAGCTATGAGAAAGCGCCACGCTTCCCGAAGGGAGAAAGGCGGACAGGTATCCGGTAAGCGGCAGGGTCGGAACAGGAGAGCGCACGAGGGAGCTTCCAGGGGGAAACGCCTGGTATCTTTATAGTCCTGTCGGGTTTCGCCACCTCTGACTTGAGCGTCGATTTTTGTGATGCTCGTCAGGGGGGCGGAGCCTATGGAAAAACGCCAGCAACGCGGCCTTTTTACGGTTCCTGGCCTTTTGCTGGCCTTTTGCTCACATGTTCTTTCCTGCGTTATCCCCTGATTCTGTGGATAACCGTATTACCGCCTTTGAGTGAGCTGATACCGCTCGCCGCAGCCGAACGACCGAGCGCAGCGAGTCAGTGAGCGAGGAAGCGGAAGAGCGCCCAATACGCAAACCGCCTCTCCCCGCGCGTTGGCCGATTCATTAATGCAGCTGGCACGACAGGTTTCCCGACTGGAAAGCGGGCAGTGAGCGCAACGCAATTAATGTGAGTTAGCTCACTCATTAGGCACCCCAGGCTTTACACTTTATGCTTCCGGCTCGTATGTTGTGTGGAATTGTGAGCGGATAACAATTTCACACAGGAAACAGCTATGACCATGATTACGCCAAGCTCGAAATTAACCCTCACTAAAGGGAACAAAAGCTGGAGCTCCACCGCGGTGGCGGCCGCTCTAGAACTAGTGGATCCCCCGGGCTGCAGGAATTCGATAGTATTATTCCGTGATACGGATACTGTAAGATACAGATGTAATACATCGTTTCTAATAGAAACTGTATCTTACAGTATCCGTATCACGGAATAATACTTTTTATATGGATTATGGACTTATATCAAATTCCTATATGGATCACTGGAGGTGGAAAATAAGAGAAAAACATAAGGTGGAAATTAAGCTTATTCTCCACACACAAATACAACCTTAATTTCCACTACCACATGGTCCACCCCTATGGATCACTGGAGGTGGAAAATAAGAAAAAAACATAAGGTGGAAATTAAGCTTATTCTCCACACACAAATACAACCTTAATTTCCACTACCACATGGTCCACCCCTATATAAGGAGTACAAAAGGAGAGGCGAATTGAGTGATGAATTCAGTCTGCGATGAACATTCGCCGTGTGAACACGGAAACTTATATTGTGAGTGCATATACTGTTGGGAACATGACGGACAGTGCAGGGGGAAAAAACTGGATTTGGGAGAATCAACTGGAATCGAAGGAAGATTGGCCAACGATAACCAACAACCAGGGCTCTCAGATTCATATTGCACCGAGACAATACATCTTGCAACTGCAGTTCAACCGGAAAGGAGAGTCATCGATCGAGAAGATTACGTCAAGAATTTCGCTGGTCAAACCGTTGGTGACCTCTACCCACAATTACAAGGCAGCACCGGAGCCTCTGAACCAATTGATTTCGCATTTCCAACTGTTGGCTCAGGAAGCTGGGAAATACTTGTACGTGAATCTCACAAACATTTCGAGCCAAATAATTCGGAAGAAGCTTATCAATCACATATTAGAAGTGTACGAAGACGATTATTCCCCGAAGAAACTATGGATAATAACGGGTCACAGGCAAGCACGACCGAAATGTTACGAAACGCTGTCGAAAGATGCGGTTTTGAAGGCCCTCCTAACAGCCCAAGCGAAAATAACAGAGATGGAGTTGATGGAACGTGTATATCAACCGTGGAATTACACAGCAATTGTATTGTTAACGCACATTGCCCAAAACAAGGACCAAGCAATCAAACCAATAAGAGAAAGAAATCAACCGATACAACAGAATCAAGCGGATCCAAAAAAAATAAAAGCAGCAATAATCAACAAAATATACAAGAACAAGGCAGTTCCAGCATCACCGACAATATCGATCTCGTCGACGGAGAGTTGGATGGATCAATTGGATCGAATCGAGAAACAGCATACTACACATTCGTCCTCCACAAAGACAACGTTAAAGAGGACTGGAGATACATCGCCACAACCAGGGCCAAGCAAGCGCCGAGTTTCATCACATTCGATCACGGAGACCACATCCATATCCTCTTCTCCTCGTCCAATACAGGAGGAAACAGCACAAGAGTCAGAACCAGAATCACCAAGTTTCTTAGTGCGACAAGCGCAGGAAGTGCAGAAGCGACTATCACTTTTTCCAAAGTTAAATTTCTCAGGAACTTCATTCTCTATTGCATCCGTTACGGTATCGAAACAGTCAATATCTATGGAAATAAAATCCAACAACAATTAACCGAAGCAATGGATACATTTAAAATATTATTTGAAAATAGAGACCCAAATGACGTAATATTAGAAGCCGGATGCAAACTATATCATGAAGAAAAAAAGGATAATAAACAAAAAAGATGCGGACAACGTAAACAACAAAATCTAACGGAAATTATATTGGAAAAAATTAAAGAAAAGAAAATAACAACGGCACAGCAATGGGAAAATCAAATTGAACCTGAATTCAAAATACAATTAATAAAAGAGTTTGGATTAAATGTAGACAGTTATGTAACAAGAATAGTACGCATCGAGAGGACACGTATACAACAGTTGATAAAAGCAAAAACGCTTACGGAAATAATGCTTGAAACATTAAATGATGACTATATTAAACACTTCACACCAGGAGAAGACAACAGTAAAACAACAAAATGTATTGAATGGATAGAATATCTATTCAAAGAAAATAACATTAATATAATCCACTTCTTGGCATGGAATGAAATTATAAAAACAAAAAGGTATAAAAAAATAAATGGAATGGTACTAGAGGGCATCACAAATGCAGGAAAATCACTAATATTGGATAACTTATTGGCCATGGTAAAACCAGAAGAAATACCACGAGAACGAGACAACAGTGGATTCCACCTTGACCAAGTACCAGGAGCAGGATCGATCCTATTTGAAGAACCAATGATCACACCAGTAAACGTCGGAACATGGAAATTATTACTAGAAGGAAAAACCATAAAAACAGATGTGAAAAATAAAGACAAAGAGCCGATAGAACGCACACCAACGTGGATCACAACAGCAACTCCAATTACAAATAACATTGATATGAATGAGACATCACAAATACTACAAAGAATAAAATTATATATATTCAAAAAAAGTATCCAACACAGAGACGACAAATATACTATAAATGCGCAAATTCAAAATAAATTGATCAGTCGTCCTCCAACTCTCATTGAGCCAATACATATGGCCATAGTGTTTATAAAAAATTTCACAAAAATATATAATCTAATCGCAGAAGAAGACAAGGCACACACAGTGAATGAAAAGGCAATACAAATAAGCAACGAAGTGAAAGAAGAAGCAGAATTATGGCAGACAGCACTTCAATGGACCATGATGGAGAACAACGAGGAACAAAACGAAAACGAGGCGCAGAAGGATCAGGTGCAGGAATCGGAAAAGGAAATAGCAACTCCGTAAAAGAAGGATATGGACCAAATATGACCGAAATGATCCCAAGAAATATTATGAACAAAGGAAATCATACGGTACTTCATACAGTAAAGCAGCAAAAATACTTGGACTTCAATTTCGTATCGAATCAAAACCCATACATTATACCATATCAAACGGCAGGATTCTGGGCATCAATGTGGGACCAAGGAGAAATCGAATCAAACAACACCATCAATATCATGAAAGCACTAAATAGAGTAGCTCTTGGAGTAACATGGATCAAAGGAGAAATCACATTCGAAGTATATTCAGTAACCAGACAACGCTTGCTAACGGGAACGACAAACCAAACTACATGGGACTTTGAAACAAGTCAAAACATGTTCATCGCAGACGCAGACAGAGAACCAGAAAACTTCGGATTAACATCAGCAGCAGCAACTGGTCCACTTGCTCAACAAACAACACAAACACTACTATTCAACAAAAACAACGACAGATACACAAAATATGAATTACCACAAAGAAATCAATATACAAGGGAAATCAACTTCCAAAAACTAACAAACAACTATATGTGGAGACCAACGGACATCAACGAAGAAGCAAACTTTAGAAGACTGATCCCAATGTCGGAAGGAGTATATACATCATCAAACGCAAATAGTAAAGCGTCAGAATTAACACAACAAAATTCAGCATGGGCTACATCAGGAAAAACAACACAAGGAACACTATTCAGAAATAGAACATCATATCCAAGAATGCATGTAGCACAACCACAAGTTCCAGATGAAACCGGATACATGAAGTTCAGATACCAAGTACGAATGAGCACAAAATTATACCTAGAATTTCATCTCTACCCAGATTATGGATCATCAACAAACATGGAATATATGCAAAGACAAGTACTGGAATTACCAGAAGTCACTGCAAGAGGAGGAGTAGTAACATGTATGCCGTATGAAATCAAAACTTAAATATTTTAATCAACATGTATCAACTATAACACATATATAATCAATAAAGCATTCAAAAAAGTATACAAGTCCAAGTCCAATTAATATATATCACAATAAAAATCCACATTAAAATATAAGCTTAATTTCCACCTCCGTAGTCCACCTCAGAATATTGGCTTAATTTCCACCTCCGTAGTCCACCTCAGAATATTGGCTTAAATTCCACCTCCGATGATACAGTTAAGAAGCCAACATTAGTCCGGGATCCCCGTGTGAGCCGATAGGCGAGGATCGAAAGCCCAAATTTTGCTGACGTCACCTCACACACATACCAAAAGCTTTAGTTTCTAATAGAAACAGCGTATTACGCTTAAAGCTTTTGGTATGTGTGTGAGGTGACGTCAGCAAAATTTGGGCTTTCGATCCTCGCCTATCGGCTATCAAGCTTATCGATACCGTCGACCTCGAGGGGGGGCCCGGTACCCAATTCGCCCTATAGTGAGTCGTATTACAATTCACTGGCCGTCGTTTTACAACGTCGTGACTGGGAAAACCCTGGCGTTACCCAACTTAATCGCCTTGCAGCACATCCCCCTTTCGCCAGCTGGCGTAATAGCGAAGAGGCCCGCACCGATCGCCCTTCCCAACAGTTGCGCAGCCTGAATGGCGAATGGAAATTGTAAGCGTTAATATTTTGTTAAAATTCGCGTTAAATTTTTGTTAAATCAGCTCATTTTTTAACCAATAGGCCGAAATCGGCAAAATCCCTTATAAATCAAAAGAATAGACCGAGATAGGGTTGAGTGTTGTTCCAGTTTGGAACAAGAGTCCACTATTAAAGAACGTGGACTCCAACGTCAAAGGGCGAAAAACCGTCTATCAGGGCGATGGCCCACTACGTGAACCATCACCCTAATCAAGTTTTTTGGGGTCGAGGTGCCGTAAAGCACTAAATCGGAACCCTAAAGGGAGCCCCCGATTTAGAGCTTGACGGGGAAAGCCGGCGAACGTGGCGAGAAAGGAAGGGAAGAAAGCGAAAGGAGCGGGCGCTAGGGCGCTGGCAAGTGTAGCGGTCACGCTGCGCGTAACCACCACACCCGCCGCGCTTAATGCGCCGCTACAGGGCGCGTCAGGTG

Full sequence of pBAgMCS (4057 bp) (yellow denotes virus insert):

GCACTTTTCGGGGAAATGTGCGCGGAACCCCTATTTGTTTATTTTTCTAAATACATTCAAATATGTATCCGCTCATGAGACAATAACCCTGATAAATGCTTCAATAATATTGAAAAAGGAAGAGTATGAGTATTCAACATTTCCGTGTCGCCCTTATTCCCTTTTTTGCGGCATTTTGCCTTCCTGTTTTTGCTCACCCAGAAACGCTGGTGAAAGTAAAAGATGCTGAAGATCAGTTGGGTGCACGAGTGGGTTACATCGAACTGGATCTCAACAGCGGTAAGATCCTTGAGAGTTTTCGCCCCGAAGAACGTTTTCCAATGATGAGCACTTTTAAAGTTCTGCTATGTGGCGCGGTATTATCCCGTATTGACGCCGGGCAAGAGCAACTCGGTCGCCGCATACACTATTCTCAGAATGACTTGGTTGAGTACTCACCAGTCACAGAAAAGCATCTTACGGATGGCATGACAGTAAGAGAATTATGCAGTGCTGCCATAACCATGAGTGATAACACTGCGGCCAACTTACTTCTGACAACGATCGGAGGACCGAAGGAGCTAACCGCTTTTTTGCACAACATGGGGGATCATGTAACTCGCCTTGATCGTTGGGAACCGGAGCTGAATGAAGCCATACCAAACGACGAGCGTGACACCACGATGCCTGTAGCAATGGCAACAACGTTGCGCAAACTATTAACTGGCGAACTACTTACTCTAGCTTCCCGGCAACAATTAATAGACTGGATGGAGGCGGATAAAGTTGCAGGACCACTTCTGCGCTCGGCCCTTCCGGCTGGCTGGTTTATTGCTGATAAATCTGGAGCCGGTGAGCGTGGGTCTCGCGGTATCATTGCAGCACTGGGGCCAGATGGTAAGCCCTCCCGTATCGTAGTTATCTACACGACGGGGAGTCAGGCAACTATGGATGAACGAAATAGACAGATCGCTGAGATAGGTGCCTCACTGATTAAGCATTGGTAACTGTCAGACCAAGTTTACTCATATATACTTTAGATTGATTTAAAACTTCATTTTTAATTTAAAAGGATCTAGGTGAAGATCCTTTTTGATAATCTCATGACCAAAATCCCTTAACGTGAGTTTTCGTTCCACTGAGCGTCAGACCCCGTAGAAAAGATCAAAGGATCTTCTTGAGATCCTTTTTTTCTGCGCGTAATCTGCTGCTTGCAAACAAAAAAACCACCGCTACCAGCGGTGGTTTGTTTGCCGGATCAAGAGCTACCAACTCTTTTTCCGAAGGTAACTGGCTTCAGCAGAGCGCAGATACCAAATACTGTCCTTCTAGTGTAGCCGTAGTTAGGCCACCACTTCAAGAACTCTGTAGCACCGCCTACATACCTCGCTCTGCTAATCCTGTTACCAGTGGCTGCTGCCAGTGGCGATAAGTCGTGTCTTACCGGGTTGGACTCAAGACGATAGTTACCGGATAAGGCGCAGCGGTCGGGCTGAACGGGGGGTTCGTGCACACAGCCCAGCTTGGAGCGAACGACCTACACCGAACTGAGATACCTACAGCGTGAGCTATGAGAAAGCGCCACGCTTCCCGAAGGGAGAAAGGCGGACAGGTATCCGGTAAGCGGCAGGGTCGGAACAGGAGAGCGCACGAGGGAGCTTCCAGGGGGAAACGCCTGGTATCTTTATAGTCCTGTCGGGTTTCGCCACCTCTGACTTGAGCGTCGATTTTTGTGATGCTCGTCAGGGGGGCGGAGCCTATGGAAAAACGCCAGCAACGCGGCCTTTTTACGGTTCCTGGCCTTTTGCTGGCCTTTTGCTCACATGTTCTTTCCTGCGTTATCCCCTGATTCTGTGGATAACCGTATTACCGCCTTTGAGTGAGCTGATACCGCTCGCCGCAGCCGAACGACCGAGCGCAGCGAGTCAGTGAGCGAGGAAGCGGAAGAGCGCCCAATACGCAAACCGCCTCTCCCCGCGCGTTGGCCGATTCATTAATGCAGCTGGCACGACAGGTTTCCCGACTGGAAAGCGGGCAGTGAGCGCAACGCAATTAATGTGAGTTAGCTCACTCATTAGGCACCCCAGGCTTTACACTTTATGCTTCCGGCTCGTATGTTGTGTGGAATTGTGAGCGGATAACAATTTCACACAGGAAACAGCTATGACCATGATTACGCCAAGCTCGAAATTAACCCTCACTAAAGGGAACAAAAGCTGGAGCTCCACCGCGGTGGCGGCCGCTCTAGAACTAGTGGATCCCCCGGGCTGCAGGAATTCGATAGTATTATTCCGTGATACGGATACTGTAAGATACAGATGTAATACATCGTTTCTAATAGAAACTGTATCTTACAGTATCCGTATCACGGAATAATACTTTTTATATGGATTATGGACTTATATCAAATTCCTATATGGATCACTGGAGGTGGAAAATAAGAGAAAAACATAAGGTGGAAATTAAGCTTATTCTCCACACACAAATACAACCTTAATTTCCACTACCACATGGTCCACCCCTATGGATCACTGGAGGTGGAAAATAAGAAAAAAACATAAGGTGGAAATTAAGCTTATTCTCCACACACAAATACAACCTTAATTTCCACTACCACATGGTCCACCCCTATATAAGGCAACTGGAATCGAAGGAAGATTGGCCAACGATAACCAACAACCAGGGCTCTCAGATTCATATTGCACCGAGACAATACAAGATCTGATATCACGCGTCCATGGATGCATGTAGCACAACCACAAGTTCCAGATGAAACCGGATACATGAAGTTCAGATACCAAGTACGAATGAGCACAAAATTATACCTAGAATTTCATCTCTACCCAGATTATGGATCATCAACAAACATGGAATATATGCAAAGACAAGTACTGGAATTACCAGAAGTCACTGCAAGAGGAGGAGTAGTAACATGTATGCCGTATGAAATCAAAACTTAAATATTTTAATCAACATGTATCAACTATAACACATATATAATCAATAAAGCATTCAAAAAAGTATACAAGTCCAAGTCCAATTAATATATATCACAATAAAAATCCACATTAAAATATAAGCTTAATTTCCACCTCCGTAGTCCACCTCAGAATATTGGCTTAATTTCCACCTCCGTAGTCCACCTCAGAATATTGGCTTAAATTCCACCTCCGATGATACAGTTAAGAAGCCAACATTAGTCCGGGATCCCCGTGTGAGCCGATAGGCGAGGATCGAAAGCCCAAATTTTGCTGACGTCACCTCACACACATACCAAAAGCTTTAGTTTCTAATAGAAACAGCGTATTACGCTTAAAGCTTTTGGTATGTGTGTGAGGTGACGTCAGCAAAATTTGGGCTTTCGATCCTCGCCTATCGGCTATCAAGCTTATCGATACCGTCGACCTCGAGGGGGGGCCCGGTACCCAATTCGCCCTATAGTGAGTCGTATTACAATTCACTGGCCGTCGTTTTACAACGTCGTGACTGGGAAAACCCTGGCGTTACCCAACTTAATCGCCTTGCAGCACATCCCCCTTTCGCCAGCTGGCGTAATAGCGAAGAGGCCCGCACCGATCGCCCTTCCCAACAGTTGCGCAGCCTGAATGGCGAATGGAAATTGTAAGCGTTAATATTTTGTTAAAATTCGCGTTAAATTTTTGTTAAATCAGCTCATTTTTTAACCAATAGGCCGAAATCGGCAAAATCCCTTATAAATCAAAAGAATAGACCGAGATAGGGTTGAGTGTTGTTCCAGTTTGGAACAAGAGTCCACTATTAAAGAACGTGGACTCCAACGTCAAAGGGCGAAAAACCGTCTATCAGGGCGATGGCCCACTACGTGAACCATCACCCTAATCAAGTTTTTTGGGGTCGAGGTGCCGTAAAGCACTAAATCGGAACCCTAAAGGGAGCCCCCGATTTAGAGCTTGACGGGGAAAGCCGGCGAACGTGGCGAGAAAGGAAGGGAAGAAAGCGAAAGGAGCGGGCGCTAGGGCGCTGGCAAGTGTAGCGGTCACGCTGCGCGTAACCACCACACCCGCCGCGCTTAATGCGCCGCTACAGGGCGCGTCAGGTG

Full Sequence of pAgActinGFP (7241 bp) (yellow denotes virus insert):

GCACTTTTCGGGGAAATGTGCGCGGAACCCCTATTTGTTTATTTTTCTAAATACATTCAAATATGTATCCGCTCATGAGACAATAACCCTGATAAATGCTTCAATAATATTGAAAAAGGAAGAGTATGAGTATTCAACATTTCCGTGTCGCCCTTATTCCCTTTTTTGCGGCATTTTGCCTTCCTGTTTTTGCTCACCCAGAAACGCTGGTGAAAGTAAAAGATGCTGAAGATCAGTTGGGTGCACGAGTGGGTTACATCGAACTGGATCTCAACAGCGGTAAGATCCTTGAGAGTTTTCGCCCCGAAGAACGTTTTCCAATGATGAGCACTTTTAAAGTTCTGCTATGTGGCGCGGTATTATCCCGTATTGACGCCGGGCAAGAGCAACTCGGTCGCCGCATACACTATTCTCAGAATGACTTGGTTGAGTACTCACCAGTCACAGAAAAGCATCTTACGGATGGCATGACAGTAAGAGAATTATGCAGTGCTGCCATAACCATGAGTGATAACACTGCGGCCAACTTACTTCTGACAACGATCGGAGGACCGAAGGAGCTAACCGCTTTTTTGCACAACATGGGGGATCATGTAACTCGCCTTGATCGTTGGGAACCGGAGCTGAATGAAGCCATACCAAACGACGAGCGTGACACCACGATGCCTGTAGCAATGGCAACAACGTTGCGCAAACTATTAACTGGCGAACTACTTACTCTAGCTTCCCGGCAACAATTAATAGACTGGATGGAGGCGGATAAAGTTGCAGGACCACTTCTGCGCTCGGCCCTTCCGGCTGGCTGGTTTATTGCTGATAAATCTGGAGCCGGTGAGCGTGGGTCTCGCGGTATCATTGCAGCACTGGGGCCAGATGGTAAGCCCTCCCGTATCGTAGTTATCTACACGACGGGGAGTCAGGCAACTATGGATGAACGAAATAGACAGATCGCTGAGATAGGTGCCTCACTGATTAAGCATTGGTAACTGTCAGACCAAGTTTACTCATATATACTTTAGATTGATTTAAAACTTCATTTTTAATTTAAAAGGATCTAGGTGAAGATCCTTTTTGATAATCTCATGACCAAAATCCCTTAACGTGAGTTTTCGTTCCACTGAGCGTCAGACCCCGTAGAAAAGATCAAAGGATCTTCTTGAGATCCTTTTTTTCTGCGCGTAATCTGCTGCTTGCAAACAAAAAAACCACCGCTACCAGCGGTGGTTTGTTTGCCGGATCAAGAGCTACCAACTCTTTTTCCGAAGGTAACTGGCTTCAGCAGAGCGCAGATACCAAATACTGTCCTTCTAGTGTAGCCGTAGTTAGGCCACCACTTCAAGAACTCTGTAGCACCGCCTACATACCTCGCTCTGCTAATCCTGTTACCAGTGGCTGCTGCCAGTGGCGATAAGTCGTGTCTTACCGGGTTGGACTCAAGACGATAGTTACCGGATAAGGCGCAGCGGTCGGGCTGAACGGGGGGTTCGTGCACACAGCCCAGCTTGGAGCGAACGACCTACACCGAACTGAGATACCTACAGCGTGAGCTATGAGAAAGCGCCACGCTTCCCGAAGGGAGAAAGGCGGACAGGTATCCGGTAAGCGGCAGGGTCGGAACAGGAGAGCGCACGAGGGAGCTTCCAGGGGGAAACGCCTGGTATCTTTATAGTCCTGTCGGGTTTCGCCACCTCTGACTTGAGCGTCGATTTTTGTGATGCTCGTCAGGGGGGCGGAGCCTATGGAAAAACGCCAGCAACGCGGCCTTTTTACGGTTCCTGGCCTTTTGCTGGCCTTTTGCTCACATGTTCTTTCCTGCGTTATCCCCTGATTCTGTGGATAACCGTATTACCGCCTTTGAGTGAGCTGATACCGCTCGCCGCAGCCGAACGACCGAGCGCAGCGAGTCAGTGAGCGAGGAAGCGGAAGAGCGCCCAATACGCAAACCGCCTCTCCCCGCGCGTTGGCCGATTCATTAATGCAGCTGGCACGACAGGTTTCCCGACTGGAAAGCGGGCAGTGAGCGCAACGCAATTAATGTGAGTTAGCTCACTCATTAGGCACCCCAGGCTTTACACTTTATGCTTCCGGCTCGTATGTTGTGTGGAATTGTGAGCGGATAACAATTTCACACAGGAAACAGCTATGACCATGATTACGCCAAGCTCGAAATTAACCCTCACTAAAGGGAACAAAAGCTGGAGCTCCACCGCGGTGGCGGCCGCTCTAGAACTAGTGGATCCCCCGGGCTGCAGGAATTCGATAGTATTATTCCGTGATACGGATACTGTAAGATACAGATGTAATACATCGTTTCTAATAGAAACTGTATCTTACAGTATCCGTATCACGGAATAATACTTTTTATATGGATTATGGACTTATATCAAATTCCTATATGGATCACTGGAGGTGGAAAATAAGAGAAAAACATAAGGTGGAAATTAAGCTTATTCTCCACACACAAATACAACCTTAATTTCCACTACCACATGGTCCACCCCTATGGATCACTGGAGGTGGAAAATAAGAAAAAAACATAAGGTGGAAATTAAGCTTATTCTCCACACACAAATACAACCTTAATTTCCACTACCACATGGTCCACCCCTATATAAGGCAACTGGAATCGAAGGAAGATTGGCCAACGATAACCAACAACCAGGGCTCTCAGATTCATATTGCACCGAGACAATACAAGATCTGATATCcgatcgctccattcttggctatatgtttttcaccgttacccggggccattttcaaagactcgtcggcaagataagattgtgtcactcgctgtctctcttcatttgtcgaagaatgctgaggaatttcgcgatgacgtcggcgagtattttgaagaatgagaataatttgtatttatacgaaaatcagttagtggaattttctacaaaaacatgttatctatagataattttgttgcaaaatatgttgactatgacaaagattgtatgtatatacctttaatgtattctcattttcttatgtatttataatggcaatgatgatactgatgatattttaagatgatgccagaccaaaaggcttgaatttctgcgtcttttgccgaacgcagtgcatgtgcaattgttgttttttggaatattcaattttcggactgtccgctttgatttcagtttcttggcttattcaaaaagcaaagtaaagccaaaaaagcgagatggcaataccaaatgcggcaaaacggtagtggaaggaaaggggtgcggggcagcggaaggaagggtggggcggggcgtggcggggtctgtggctgggcgcgacgtcaccgacgttggagccactcctttgaccatgtgtgcgtgtgtgtattattcgtgtctcgccactcgccggttgtttttttctttttatgctgcgctctctctagcgccatctcgcttacgcatgctcaacgcaccgcatgttgccgtttccttttatgcgtcattttggctcgaaataggcaattatttaaacaaagattagtcaacgaaaacgctaaaataaataagtctacaatatggttacttattgccatgtgtgtgcagccaacgatagcaacaaaagcaacaacacaggtggctttccctctttcactttttgtttgcaagccgcgtgcgagcaagacggcacgaccggcaaacgcaattacgctgacaaagagcagacgaagttttggcgaaaaacatcaaggcgcctgatacgaatgcatttgcaataacaattgcgatatttaatattgtttatgaagctgtttgacttcaaaacacacaaaaaaaaaaataaaacaaattatttgaaagagaattaggaatcggacgcttatcgttagggtaacaacaagaaatgcttactgagtcacagcctctggaaaactgccgcaagccagagagagagagaaaaagagggagagcagcttagaccgcatgtgcttgtgtgtgaggcgtctctctcttcgtctctgttgcgcaaacgcatagactgcactgaaaaaatcgattacctattttttatgaatgaatatttgcactattactattcaaaactattaagatagcaatcacattcaatagccaaatactataccacctgagcgatgcaacgaaatgatcaatttgagcaaaaatgctgcatatttaggacggcatcattatagaaatgcttcttgctgtgtacttttctctcgtctggcagctgtttcgccgttattgttaaaaccggcttaagttaggtgtgttttctacgactagtgaatgccctactagaagatgtgtgttgcacaaaatgtccctggaataaccaatttgaagtgcagatagcagtaaacgtaagctaatatgaatattatttaactgtaatgttttaatatcgctggacattactaataaacccactataaacacatgtacatatgtatgttttggcatacaatgagtagttggggaaaaaatgtgtaaaagcaccgtgaccatcacagcataaagataaccagctgaagtatcgaatatgagtaacccccaaattgaatcacatgccgcaactgataggacccatggaagtacactcttcatggcgatatacaagacacacacaagcacgaacacccagttgcggaggaaattctccgtaaatgaaaacccaatcggcgaacaattcatacccatatatggtaaaagttttgaacgcgacttgagagcggagagcattgcggctgataaggttttagcgctaagcgggctttataaaacgggctgcgggaccagttttcatatcactaccgtttgagttcttgtgctgtgtggatactcctcccgacacaaagccgctccatcagccagcagtcgtctaatccagagaccccggatccaccggtcgccaccatggtgagcaagggcgaggagctgttcaccggggtggtgcccatcctggtcgagctggacggcgacgtaaacggccacaagttcagcgtgtccggcgagggcgagggcgatgccacctacggcaagctgaccctgaagttcatctgcaccaccggcaagctgcccgtgccctggcccaccctcgtgaccaccctgacctacggcgtgcagtgcttcagccgctaccccgaccacatgaagcagcacgacttcttcaagtccgccatgcccgaaggctacgtccaggagcgcaccatcttcttcaaggacgacggcaactacaagacccgcgccgaggtgaagttcgagggcgacaccctggtgaaccgcatcgagctgaagggcatcgacttcaaggaggacggcaacatcctggggcacaagctggagtacaactacaacagccacaacgtctatatcatggccgacaagcagaagaacggcatcaaggtgaacttcaagatccgccacaacatcgaggacggcagcgtgcagctcgccgaccactaccagcagaacacccccatcggcgacggccccgtgctgctgcccgacaaccactacctgagcacccagtccgccctgagcaaagaccccaacgagaagcgcgatcacatggtcctgctggagttcgtgaccgccgccgggatcactctcggcatggacgagctgtacaagtaaagcggccgcgactctagatcataatcagccataccacatttgtagaggttttacttgctttaaaaaacctcccacacctccccctgaacctgaaacataaaatgaatgcaattgttgttgttaacttgtttattgcagcttataatggttacaaataaagcaatagcatcacaaatttcacaaataaagcatttttttcactgcattctagttgtggtttgtccaaactcatcaatgtatcttaaggcgtaaattgtaagcgGATATCACGCGTCCATGGATGCATGTAGCACAACCACAAGTTCCAGATGAAACCGGATACATGAAGTTCAGATACCAAGTACGAATGAGCACAAAATTATACCTAGAATTTCATCTCTACCCAGATTATGGATCATCAACAAACATGGAATATATGCAAAGACAAGTACTGGAATTACCAGAAGTCACTGCAAGAGGAGGAGTAGTAACATGTATGCCGTATGAAATCAAAACTTAAATATTTTAATCAACATGTATCAACTATAACACATATATAATCAATAAAGCATTCAAAAAAGTATACAAGTCCAAGTCCAATTAATATATATCACAATAAAAATCCACATTAAAATATAAGCTTAATTTCCACCTCCGTAGTCCACCTCAGAATATTGGCTTAATTTCCACCTCCGTAGTCCACCTCAGAATATTGGCTTAAATTCCACCTCCGATGATACAGTTAAGAAGCCAACATTAGTCCGGGATCCCCGTGTGAGCCGATAGGCGAGGATCGAAAGCCCAAATTTTGCTGACGTCACCTCACACACATACCAAAAGCTTTAGTTTCTAATAGAAACAGCGTATTACGCTTAAAGCTTTTGGTATGTGTGTGAGGTGACGTCAGCAAAATTTGGGCTTTCGATCCTCGCCTATCGGCTATCAAGCTTATCGATACCGTCGACCTCGAGGGGGGGCCCGGTACCCAATTCGCCCTATAGTGAGTCGTATTACAATTCACTGGCCGTCGTTTTACAACGTCGTGACTGGGAAAACCCTGGCGTTACCCAACTTAATCGCCTTGCAGCACATCCCCCTTTCGCCAGCTGGCGTAATAGCGAAGAGGCCCGCACCGATCGCCCTTCCCAACAGTTGCGCAGCCTGAATGGCGAATGGAAATTGTAAGCGTTAATATTTTGTTAAAATTCGCGTTAAATTTTTGTTAAATCAGCTCATTTTTTAACCAATAGGCCGAAATCGGCAAAATCCCTTATAAATCAAAAGAATAGACCGAGATAGGGTTGAGTGTTGTTCCAGTTTGGAACAAGAGTCCACTATTAAAGAACGTGGACTCCAACGTCAAAGGGCGAAAAACCGTCTATCAGGGCGATGGCCCACTACGTGAACCATCACCCTAATCAAGTTTTTTGGGGTCGAGGTGCCGTAAAGCACTAAATCGGAACCCTAAAGGGAGCCCCCGATTTAGAGCTTGACGGGGAAAGCCGGCGAACGTGGCGAGAAAGGAAGGGAAGAAAGCGAAAGGAGCGGGCGCTAGGGCGCTGGCAAGTGTAGCGGTCACGCTGCGCGTAACCACCACACCCGCCGCGCTTAATGCGCCGCTACAGGGCGCGTCAGGTG
